# Current Scientific Impact of Ss Cyril and Methodius University of Skopje, Republic of Macedonia in the Scopus Database (1960-2014)

**DOI:** 10.3889/oamjms.2015.019

**Published:** 2015-01-26

**Authors:** Mirko Spiroski

**Affiliations:** Institute of Immunobiology and Human Genetics, Faculty of Medicine, Ss Cyril and Methodius University of Skopje, Skopje, Republic of Macedonia

**Keywords:** Scopus database, citation metrics, h-index, Ss Cyril and Methodius University of Skopje, Republic of Macedonia

## Abstract

**AIM:**

The aim of this study was to analyze current scientific impact of Ss Cyril and Methodius University of Skopje, Republic of Macedonia in the Scopus Database (1960-2014).

**MATERIAL AND METHODS:**

Affiliation search of the Scopus database was performed on November 23, 2014 in order to identify published papers from the Ss Cyril and Methodius University of Skopje (UC&M), Republic of Macedonia. A total number of 3960 articles (3055 articles from UC&M, 861 articles from Faculty of Medicine, UC&M, and 144 articles from Faculty of Pharmacy, UC&M) were selected for analysis (1960-2014). SCImago Journal Rank (SJR), Source Normalized Impact per Paper (SNIP) and h-index were calculated from the Scopus database.

**RESULTS:**

The number of published papers was sharply increased with maximum of 379 papers in 2012 year. The largest number of papers has been published in Macedonian Journal of Medical Sciences, Journal of Molecular Structure, Lecture Notes in Computer Science, Acta Pharmecutica, and Macedonian Journal of Chemistry and Chemical Engineering. The biggest SJR and SNIP has journal Nephrology Dialysis Transplantation. First three places of the top ten authors belong to Dimirovski GM, Gavrilovska L, and Gusev M. Top three places based on Scopus h-index (total number of published papers) belong to Kocarev L, Stafilov T, and Polenakovic M. The majority of papers originate from UC&M, but significant numbers of papers are affiliated to Faculty of Medicine, Faculty of Pharmacy, and Institute of Chemistry as members of UC&M, as well as Macedonian Academy of Sciences and Arts. Articles are the most dominant type of documents followed by conference papers, and review articles. Medicine is the most represented subject.

**CONCLUSION:**

Officials of the Ss Cyril and Methodius University of Skopje should undertake more effective and proactive policies for journal publishers and their Editorial Boards in order to include more journals from UC&M in the Scopus database.

## Introduction

Scopus is the world’s largest abstract and citation database of peer-reviewed literature with smart tools that track, analyze and visualize research. Easy to use and comprehensive, Scopus is designed to quickly find the information researchers’ need. Scopus indexes over 20,500 titles from 5,000 publishers worldwide; contains 49 million records, 78% with abstracts; includes over 5.3 million conference papers; and provides 100% Medline coverage [1]. Scopus includes a more expanded spectrum of journals than PubMed and Web of Science, and its citation analysis is faster and includes more articles than the citation analysis of Web of Science. On the other hand, the citation analysis that Web of Science presents provides better graphics and is more detailed than the citation analysis of Scopus, probably because Web of Science has been designed with the intention of satisfying users in citation analysis, a field discussed and debated by scientists for decades [2].

The three databases (WoS, Scopus and Google Scholar) represent different approaches to citation search services. WoS and Scopus are commercial databases (at the expensive end of the spectrum – for good reasons). Google Scholar is currently an open access database, still in beta version after its launch in November 2004. The expectations are different for fee-based and free databases, but open access should not provide excuse for ill-conceived and poorly implemented search options, and for convoluted, and potentially misleading presentation of information [3].

There are several rankings of the universities in the world which uses different data for analysis. Academic Ranking of World Universities (ARWU), also known as the Shanghai Ranking, is annual university rankings published by Shanghai Ranking Consultancy [4]. The publication now comprises world’s overall and subject league tables, together with regional Greater China Ranking and Macedonian HEIs Ranking. The Academic Ranking of World Universities (ARWU) compiled Macedonian HEIs Ranking, a ranking of Macedonian Higher Education Institutions (HEIs) commissioned by Ministry of Education and Science of Republic of Macedonia. Nineteen qualified HEIs were included in the ranking. The ranking used 19 indicators of academic performance and competitiveness, covering major mission aspects of HEIs such as teaching, research and social service. It is the first university ranking in Macedonia [5].

Every year, SCImago Research Group publishes two reports on institutions, the Ibero-American SIR (SCImago Research Group) and the Global SIR. The Global SIR is published in July and it takes into account those organizations from any country, with at least 100 documents published in the last year of the five-year period. The chronological range extends from 2003 to 2013 and each report represents the five-year period with indicators [6]. At the moment there are five public universities and fifteen private universities, or in total twenty accredited universities in R. Macedonia [7]. The oldest Public University in the Republic of Macedonia is Ss Cyril and Methodius University of Skopje, established in 1949 year.

The aim of this study was to analyze current scientific impact of Ss Cyril and Methodius University of Skopje, Republic of Macedonia in the Scopus Database (1960-2014).

## Material and Methods

Affiliation search of the Scopus database was performed on November 23, 2014 in order to identify published papers from the Ss Cyril and Methodius University of Skopje (UC&M), Republic of Macedonia. From the total of 4 affiliated institutions under the term “Ss Cyril and Methodius University”, three institutions were grouped in Skopje, Republic of Macedonia [AF-ID (“Ss Cyril and Methodius University” 60072629) OR AF-ID (“Ss Cyril and Methodius University Faculty of Medicine” 60072630) OR AF-ID (“Ss Cyril and Methodius University Faculty of Pharmacy” 60072638)] and one in Trnava, Slovakia [AF-ID (“University of SS Cyril and Methodius Trnava” 60021677)] which was excluded from analysis.

A total number of 3960 articles, all from Ss Cyril and Methodius University (3055 articles from Ss Cyril and Methodius University, 861 articles from Ss Cyril and Methodius University, Faculty of Medicine, and 144 articles from Ss Cyril and Methodius University, Faculty of Pharmacy), were selected for analysis (1960-2014). SCImago Journal Rank (SJR), Source Normalized Impact per Paper (SNIP) and h-index were calculated from the Scopus database [8, 9].

## Results

**Figure 1:**
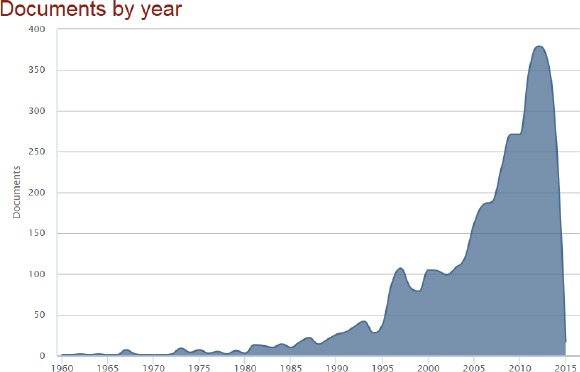
*Current distribution of published papers from the Ss Cyril and Methodius University of Skopje, Republic of Macedonia deposited in the Scopus database (1960-2014)*.

The number of publication in the period of 1960-1991 year was very small (1-29 papers per year). Starting of 1991 year, the number of published papers was sharply increased with maximum of 379 papers in 2012 year (Fig. 1).

**Figure 2:**
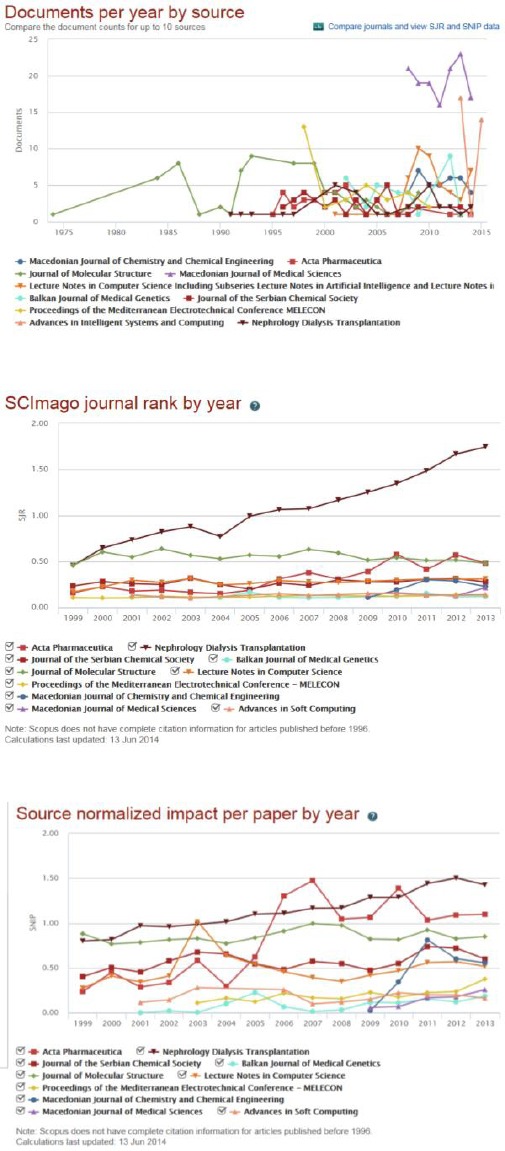
*Current documents per year by source (top), current SCImago journal rank by year (middle), and current source normalized impact per paper by year (bottom) from the Ss Cyril and Methodius University of Skopje, Republic of Macedonia in first ten journals deposited in the Scopus database (1960-2014)*.

The largest number of papers from Ss Cyril and Methodius University of Skopje have been published in Macedonian Journal of Medical Sciences (136 papers), followed by Journal of Molecular Structure (79 papers), Lecture Notes in Computer Science (46 papers), Acta Pharmecutica (38), and Macedonian Journal of Chemistry and Chemical Engineering (37 papers). The rest of journals published less than 36 papers (Fig. 2 top). The biggest SCImago journal rank per year (SJR) has journal Nephrology Dialysis Transplantation with SJR of 0.475 for 1999 year, steadily and shapely increased in the following years with the SJR of 1.744 for the 2013 year. The rest of the journals have equal or lower SJR than 0.5 with small variations and without significant increase (Fig. 2 middle). The biggest Source Normalized Impact per Paper (SNIP) has Nephrology Dialysis Transplantation (0.799 to 1.502) following by Acta Pharmaceutica and Journal of Molecular Structure (1.096 and 0.849 in 2013 year, respectively) (Fig. 2 bottom).

**Figure 3:**
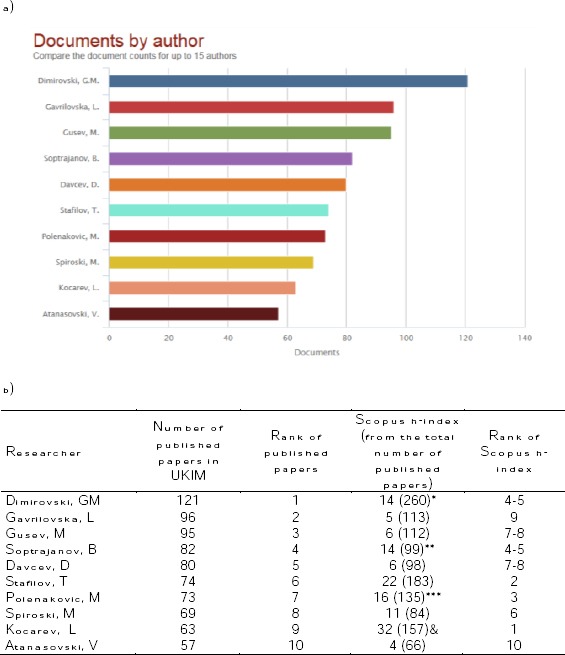
(a) Current number of published papers and (b) Hirsh index (h-index) by the top 10 authors from the Ss Cyril and Methodius University of Skopje, Republic of Macedonia in the Scopus database for 1960-2014 year. *Now affiliated at Dogus Universitesi Istanbul, School of Engineering, Istanbul, Turkey; **, additional profile with +5 papers affiliated with Hemiski Institut, Yugoslavia (not included in the first profile affiliated with Macedonian Academy of Sciences and Arts); ***, additional profile with +9 papers published in Godisen zbornik na Medicinskiot fakultet vo Skopje (not included in the first profile affiliated with Macedonian Academy of Sciences and Arts); &, additional profile with +2 papers affiliated with Ss Cyril and Methodius University of Skopje, Republic of Macedonia (not included in the first profile affiliated with Macedonian Academy of Sciences and Arts).

The number of papers published by top ten authors is shown in Figure 3. First three places belong to Dimirovski GM, Gavrilovska L, and Gusev M with 121, 96, and 95 papers, respectively. Next seven places belongs to Soptrajanov B, Davcev D, Stafilov T, Polenakovic M, Spiroski M, Kocarev L, and Atanasovski V with 82 to 52 papers. The rest of the authors affiliated to Ss Cyril and Methodius University of Skopje have published less than 52 papers each (Fig. 3).

Scopus Hirsh index (h-index) for the top ten authors affiliated to Ss Cyril and Methodius University of Skopje was presented as calculated by Scopus. We can see from the Fig. 3b that total number of published papers in Scopus database was bigger than the number of papers affiliated to Ss Cyril and Methodius University of Skopje in the same database. Top three places based on Scopus h-index (total number of published papers) belongs to Kocarev, L (h-index = 31), Stafilov, T (h-index = 22), and Polenakovic, M (h-index = 16). The rest of authors have h-index equal or lower than 14 (Fig. 3b).

**Figure 4:**
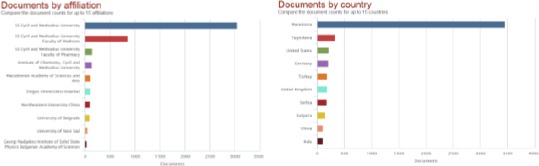
*Current documents by affiliation (left) and current documents by country (right) from the Ss Cyril and Methodius University of Skopje, Republic of Macedonia in the Scopus database (1960-2014)*.

The biggest number of papers belongs to Ss Cyril and Methodius University (3051 papers), but significant number of papers belongs to Faculty of Medicine, Ss Cyril and Methodius University (860 papers), Faculty of Pharmacy, Ss Cyril and Methodius University (145 papers), Institute of Chemistry, Cyril and Methodius University (138 papers), as well as Macedonian Academy of Sciences and Arts (105 papers) (Fig. 4 left). Most of the papers affiliated to Ss Cyril and Methodius University of Skopje originate from Macedonia (3451 papers), from Yugoslavia (336 papers), from United States (210 papers), and from Germany (207 papers). The rest of the countries participate with less than 200 papers (Fig. 4 right). The appearance of the names of other countries means that authors from the other countries are co-authors of the papers with the authors from Ss Cyril and Methodius University.

**Figure 5:**
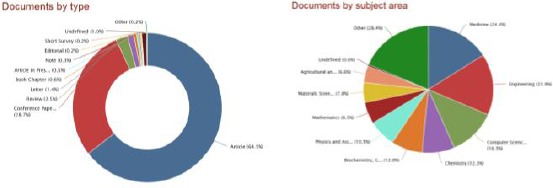
*Current percentage of documents by type (left) and current percentage of documents by subject area (right) from the Ss Cyril and Methodius University of Skopje, Republic of Macedonia in the Scopus database (1960-2014)*.

Documents by type and documents by subject area are published by Ss Cyril and Methodius University of Skopje, shown in Fig. 5. Articles are the most dominant type of documents (64.5%) followed by conference papers (28.7%), and review articles (2.5%). The rest of the documents are distributed in less than 2.0% (letters, book chapters, articles in press, notes, editorials, short surveys, undefined and others) (Fig. 5 left). Medicine is represented with 24.4% in the published papers by Ss Cyril and Methodius University of Skopje, engineering with 23.9%, computer science with 18.3%, chemistry with 12.3%, biochemistry, genetics and molecular biology with 12.0%, physics and astronomy with 10.5%. The rest of the subjects are represented in less than 10% (Fig. 5 right).

## Discussion

In this paper analysis of documents affiliated to Ss Cyril and Methodius University of Skopje deposited in the Scopus database (1960-2014) is presented. Starting of 1991 year, the number of published papers was sharply increased with maximum of 379 papers in 2012 year. The largest number of papers from Ss Cyril and Methodius University of Skopje has been published in Macedonian Journal of Medical Sciences, Journal of Molecular Structure, Lecture Notes in Computer Science, Acta Pharmecutica, and Macedonian Journal of Chemistry and Chemical Engineering. The biggest SCImago journal rank per year (SJR) and Source Normalized Impact per Paper (SNIP) has journal Nephrology Dialysis Transplantation. First three places of the top ten authors belongs to Dimirovski GM, Gavrilovska L, and Gusev M. Top three places based on Scopus h-index (total number of published papers) belongs to Kocarev L, Stafilov T, and Polenakovic M.

The majority of papers originate from Ss Cyril and Methodius University of Skopje (3051 papers from the total number of 3960 papers), but significant number of papers are affiliated to Faculty of Medicine, Faculty of Pharmacy, and Institute of Chemistry as a members of Ss Cyril and Methodius University of Skopje, as well as Macedonian Academy of Sciences and Arts. Most of the papers affiliated to Ss Cyril and Methodius University of Skopje originate from Macedonia, Yugoslavia, United States, and Germany. Articles are the most dominant type of documents followed by conference papers, and review articles. Medicine is the most represented subject. Engineering, computer science, chemistry, biochemistry, genetics and molecular biology, physics and astronomy are represented in more than 10%.

From the top 10 journals participating in the Ss Cyril and Methodius University of Skopje publishing, only two of them are published in Republic of Macedonia (Macedonian Journal of Medical Sciences and Macedonian Journal of Chemistry and Chemical Engineering). The biggest SCImago journal rank per year and the biggest Source Normalized Impact per Paper have the journal Nephrology Dialysis Transplantation, published outside the Republic of Macedonia. Thus, the impact of journals from the Ss Cyril and Methodius University of Skopje participating in Scopus database is very small. Other journals supported, attached or affiliated to mention UC&M as a publisher are not participating at all in the Scopus database. Officials of the UC&M should undertake more effective and proactive policies for journal publishers and their Editorial Boards in order to include more journals from UC&M in the Scopus database.

Analysis of author rank is very sensitive issue. In this analysis total number of published papers in Scopus database was bigger than the number of papers affiliated to Ss Cyril and Methodius University of Skopje in the same database. These differences are caused by affiliation changes of the top ten authors. For example, Dimirovski GM was affiliated with 121 papers to Ss Cyril and Methodius University of Skopje, and the rest of 139 papers are affiliated to Dogus Universitesi Istanbul, School of Engineering, Istanbul, Turkey. Similarly, Soptrajanov B, Polenakovic M, and Kocarev L have additional profiles (not calculated in this analysis) as a result of their affiliation to Macedonian Academy of Sciences and Arts. Additional problems arise when author impact analysis search is used: i) some authors have used different names in different periods of time; ii) some authors transliterate their names in several different ways; iii) some authors add or do not add “v”; iv) some authors use only one of their two surnames; v) there are some identical names and surnames in the world for different researchers [10]. All of this variability is recognized as author ambiguity. One of the effective solutions for ambiguity resolution is registration of researchers in the ResearchersID (http://www.researcherid.com/) [11] and in the ORCID (http://orcid.org/) [12]. The Scopus Author Identifier assigns a unique number to groups of documents written by the same author via an algorithm that matches authorship based on a certain criteria. If a document cannot be confidently matched with an author identifier, it is grouped separately. In this case, you may see more than 1 entry for the same author [13, 14]. Identification of authors with all their variations and papers they published (ambiguity resolution) is the sole responsibility of the authors.

For the first time in Republic of Macedonia, current scientific impact of academic staff employed at the Institutes of the Faculty of Medicine, Ss Cyril and Methodius University of Skopje, Republic of Macedonia, was analyzed and published. Based on different citation values and citation indexes, it was concluded that Institutes of the Faculty of Medicine are very heterogeneous with three groups of institutes: institutes with higher scientific impact; institutes with intermediate scientific impact, and institutes with low scientific impact [15]. Scientific impact of all institutions connected with education and research should be analyzed and available publicly in order to follow the development of academic staff and institutions in which they are affiliated.

Recently we presented the results of SCImago Institutions Rankings for R. Macedonia (2009-2012) and show that only Ss Cyril and Methodius University of Skopje were included in the SIR with world rank of 1726 to 1821. Regional rank in Eastern Europe was gradually increased from 156th in 2009 to 127th in 2013 year [7]. The results in this paper using Scopus database are similar to previously published paper and prove the fact that Ss Cyril and Methodius University of Skopje is the only University from Republic of Macedonia fulfilling the inclusion criteria for institutional analysis in SCImago Institutions Rankings.

In summary, analysis of documents affiliated to Ss Cyril and Methodius University of Skopje deposited in the Scopus database (1960-2014) have shown that the number of published papers was sharply increased with maximum of 379 papers in 2012 year. The largest number of papers from Ss Cyril and Methodius University of Skopje has been published in Macedonian Journal of Medical Sciences. The biggest SCImago journal rank per year and Source Normalized Impact per Paper has journal Nephrology Dialysis Transplantation. First three places of the top ten authors belong to Dimirovski GM, Gavrilovska L, and Gusev M. Top three places based on Scopus h-index (total number of published papers) belong to Kocarev L, Stafilov T, and Polenakovic M. Officials of the UC&M should undertake more effective and proactive policies for journal publishers and their Editorial Boards in order to include more journals from UC&M in the Scopus database.

## References

[1] Bakkalbasi N, Bauer K, Glover J, Wang L (2006). Three options for citation tracking: Google Scholar, Scopus and Web of Science. Biomed Digit Libr.

[2] Falagas ME, Pitsouni EI, Malietzis GA, Pappas G (2008). Comparison of PubMed, Scopus, Web of Science, and Google Scholar: strengths and weaknesses. FASEB J.

[3] Jacso P (2005). As we may search-Comparison of major features of the Web of Science, Scopus, and Google Scholar citation-based and citation-enhanced databases. Current Science-Bangalore.

[4] Liu NC, Cheng Y (2005). The academic ranking of world universities. Higher education in Europe.

[5] Mester G (2009/2010). Academic Ranking of World Universities.

[6] Bakkalbasi N, Bauer K, Glover J, Wang L (2006). Three options for citation tracking: Google Scholar, Scopus and Web of Science. Biomed Digit Libr.

[7] Spiroski M (2014). Current biomedical scientific impact (2013) of institutions, academic journals and researchers in the Republic of Macedonia. Prilozi.

[8] González-Pereira B, Guerrero-Bote V, Moya-Anegon F (2009). The SJR indicator: A new indicator of journals’ scientific prestige. arXiv preprint arXiv.

[9] (2007). SCImago. SJR —SCImago Journal & Country Rank.

[10] Spiroski M (2009). Who is Who-Current Scientific Impact of the Medical Staff Affiliated at the Institutes, Faculty of Medicine, University“Ss Kiril and Metodij”, Skopje, Republic of Macedonia. Maced J Med Sci.

[11] Zhaohui LHC (2010). Building a New Institutional Repository Based on the Knowledge Network of Researchers: A case of Researcher ID [J]. Researches in Library Science.

[12] Anstey A (2014). How can we be certain who authors really are? Why ORCID is important to the British Journal of Dermatology. Br J Dermatol.

[13] Tan TW, Tong JC, Khan AM, de Silva M, Lim KS, Ranganathan S (2010). Advancing standards for bioinformatics activities: persistence, reproducibility, disambiguation and Minimum Information About a Bioinformatics investigation (MIABi). BMC genomics.

[14] Strotmann A, Zhao D (2012). Author name disambiguation: What difference does it make in author-based citation analysis?. Journal of the American Society for Information Science and Technology.

[15] Spiroski M (2009). Current Scientific Impact of the Institutes, Faculty of Medicine, University “Ss Kiril and Metodij”, Skopje, Republic of Macedonia. Maced J Med Sci.

